# Supplementary figures and images for: A field-wide assessment of differential expression profiling by high-throughput sequencing reveals widespread bias
Source: PLoS Biol. 2023 Mar 2;21(3):e3002007. doi: 10.1371/journal.pbio.3002007 (PMC10013925; doi:10.1371/journal.pbio.3002007)

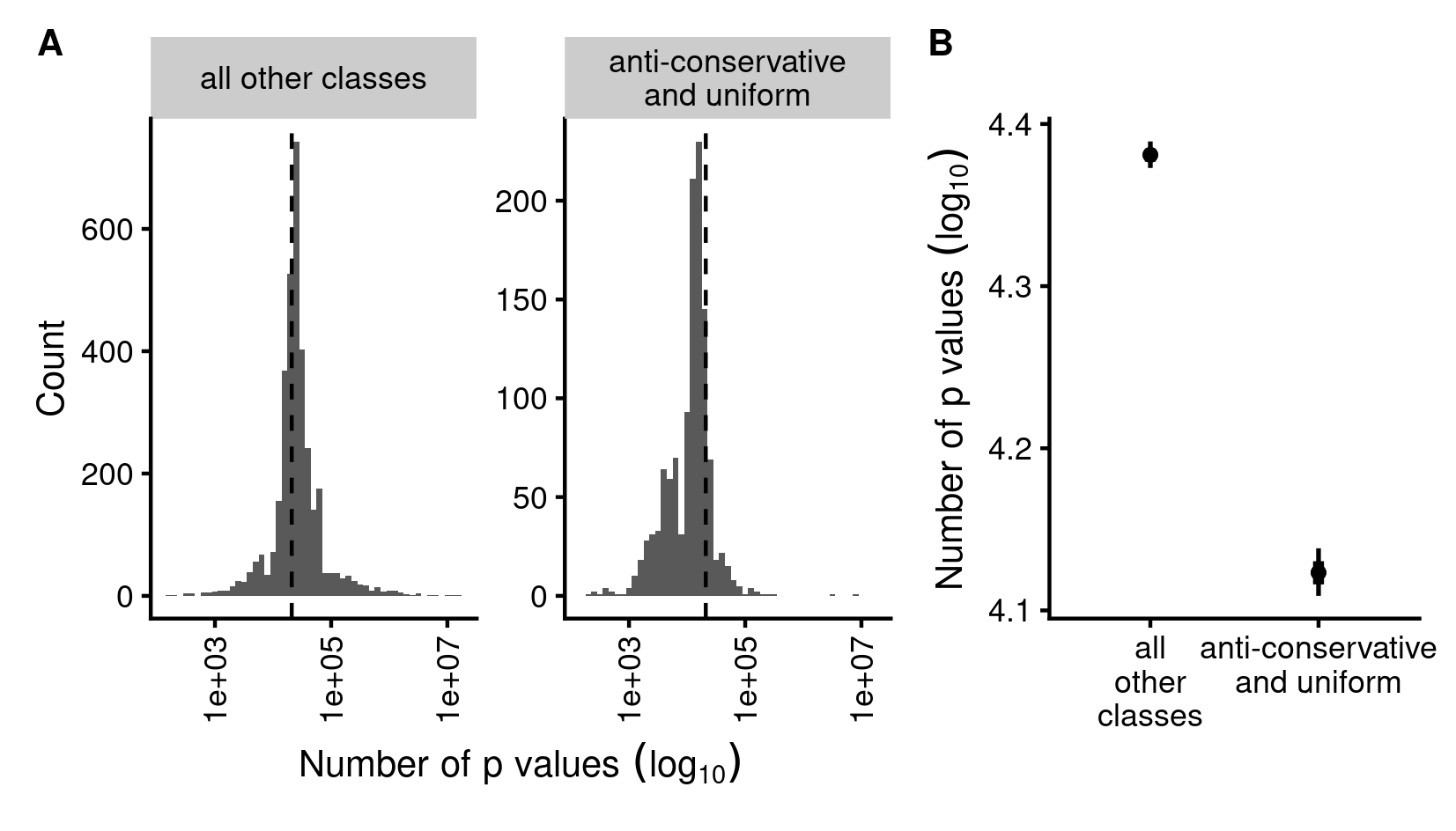

Supplement: S1 Fig — (A) P-value set size distribution. Dashed line denotes the median number of features. From each GEO series, only 1 random set was considered, N = 4,616 p-value sets. The data file is in S17 Data. (B) Robust linear modeling of number of features in anti-conservative and uniform vs. non-anti-conservative p-value sets [log10_n_pvalues ~ anticons, Student’s t likelihood], N = 4,616. Points denote best fit of linear model. Thick and thin lines denote 66% and 95% credible region, respectively. The data file is in S18 Data. The model object related to panel B can be downloaded from https://gin.g-node.org/tpall/geo-htseq-paper/src/v0.1/models/log10_n_pvalues%20~%20anticons.rds. (TIFF) [file pbio.3002007.s001.tiff]

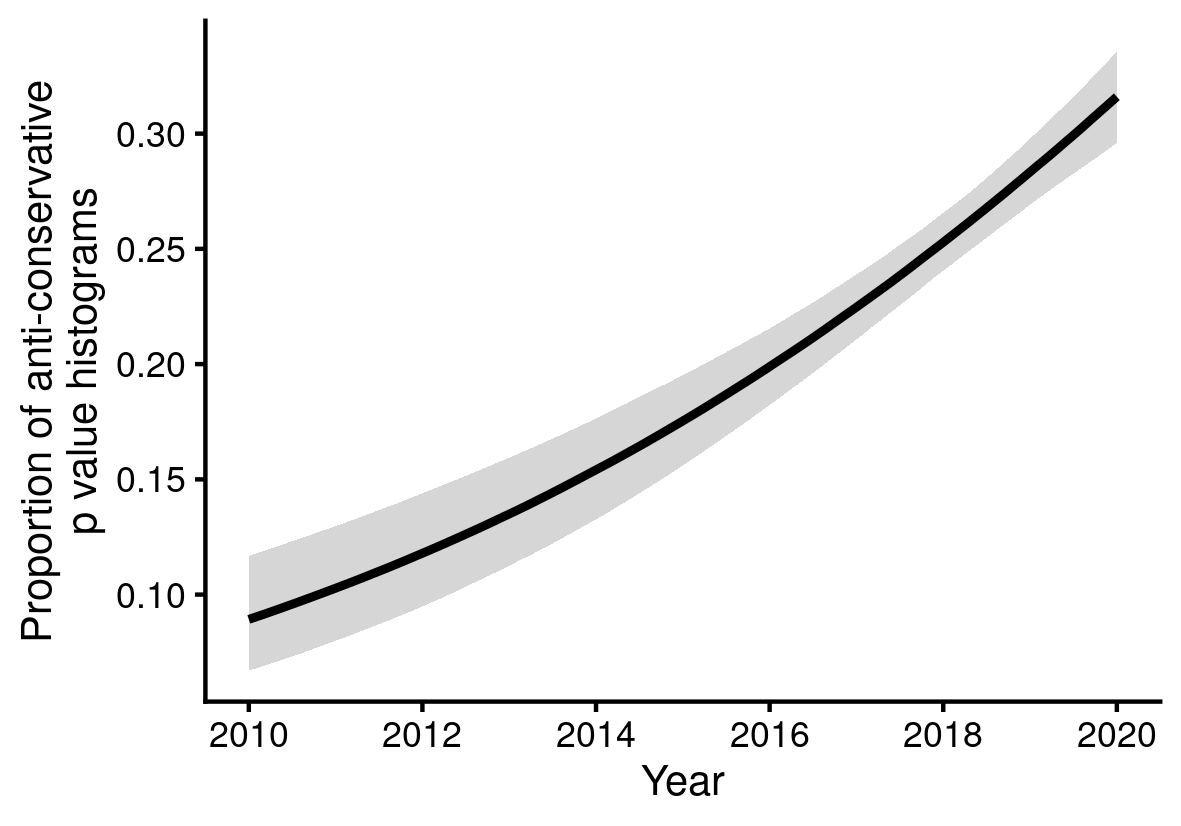

Supplement: S2 Fig — Bernoulli model [anticons ~ year], N = 4,616. Lines denote best fit of linear model. Shaded area denotes 95% credible region. The data file is in S19 Data. The model object related to figure can be downloaded from https://gin.g-node.org/tpall/geo-htseq-paper/src/v0.1/models/anticons_year.rds. (TIFF) [file pbio.3002007.s002.tiff]

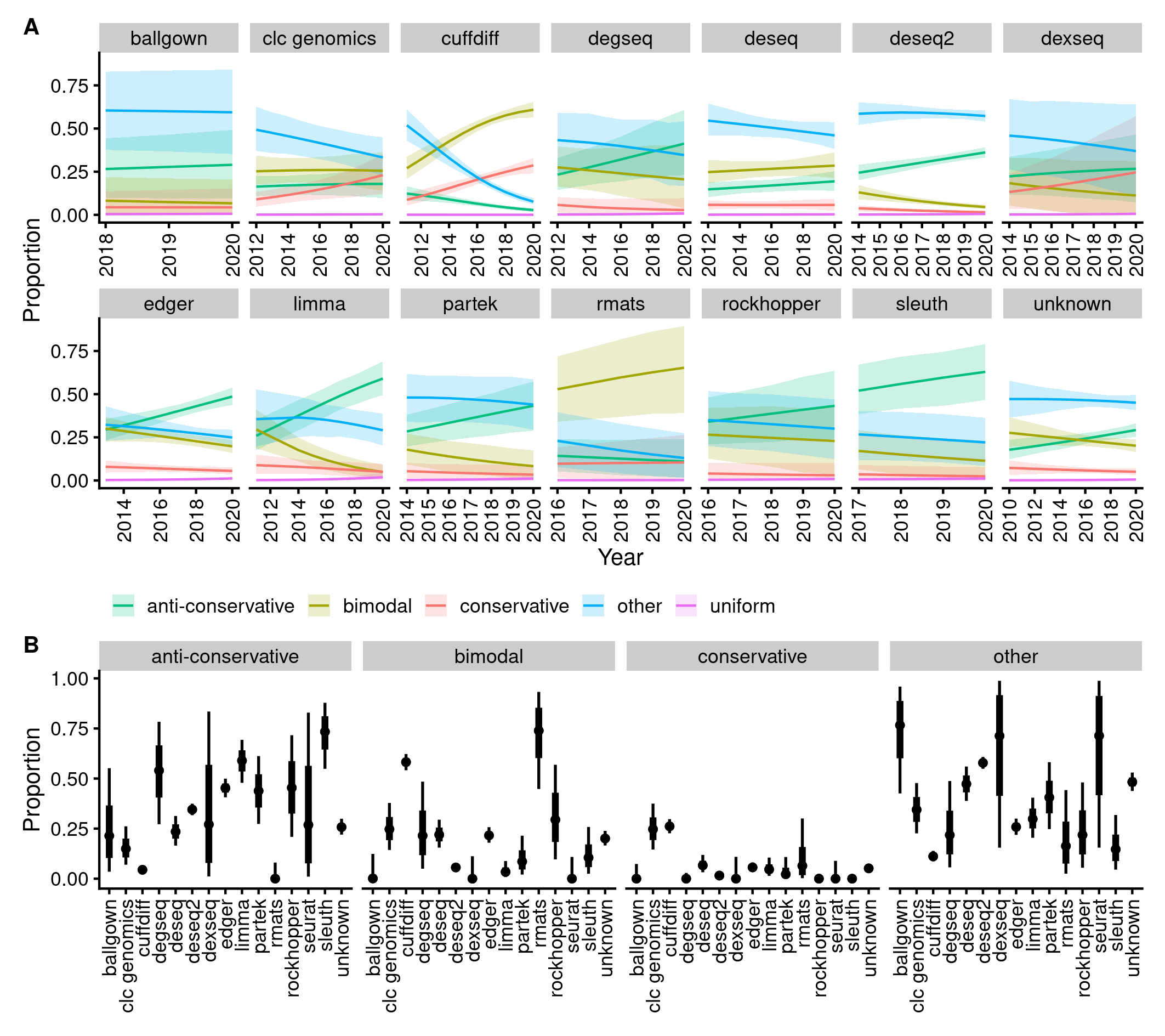

Supplement: S3 Fig — (A) Time courses for proportions of different p-value histogram classes for the 9 most frequent DE analysis platforms. Lines denote best fit of the model [Class ~ year + (year | de_tool), categorical likelihood]. Shaded areas denote 95% credible regions. N = 4,616. The data file is in S20 Data. (B) Association of p-value histogram type with DE analysis tool; data is restricted to 2018–2020 GEO submissions. Points denote best fit of the model [n | trials(total_in_de_tool) ~ Class + de_tool + Class:de_tool, binomial likelihood]. Thick and thin lines denote 66% and 95% credible intervals, respectively. N = 2,930. The data file is in S21 Data. The model object related to panel A can be downloaded from https://gin.g-node.org/tpall/geo-htseq-paper/src/v0.1/models/Class_year__year_detool_year.rds. The model object related to panel B can be downloaded from https://gin.g-node.org/tpall/geo-htseq-paper/src/v0.2/models/n__trials%28total_in_de_tool%29__Class_de_tool_Class:de_tool_2018up.rds. (TIFF) [file pbio.3002007.s003.tiff]

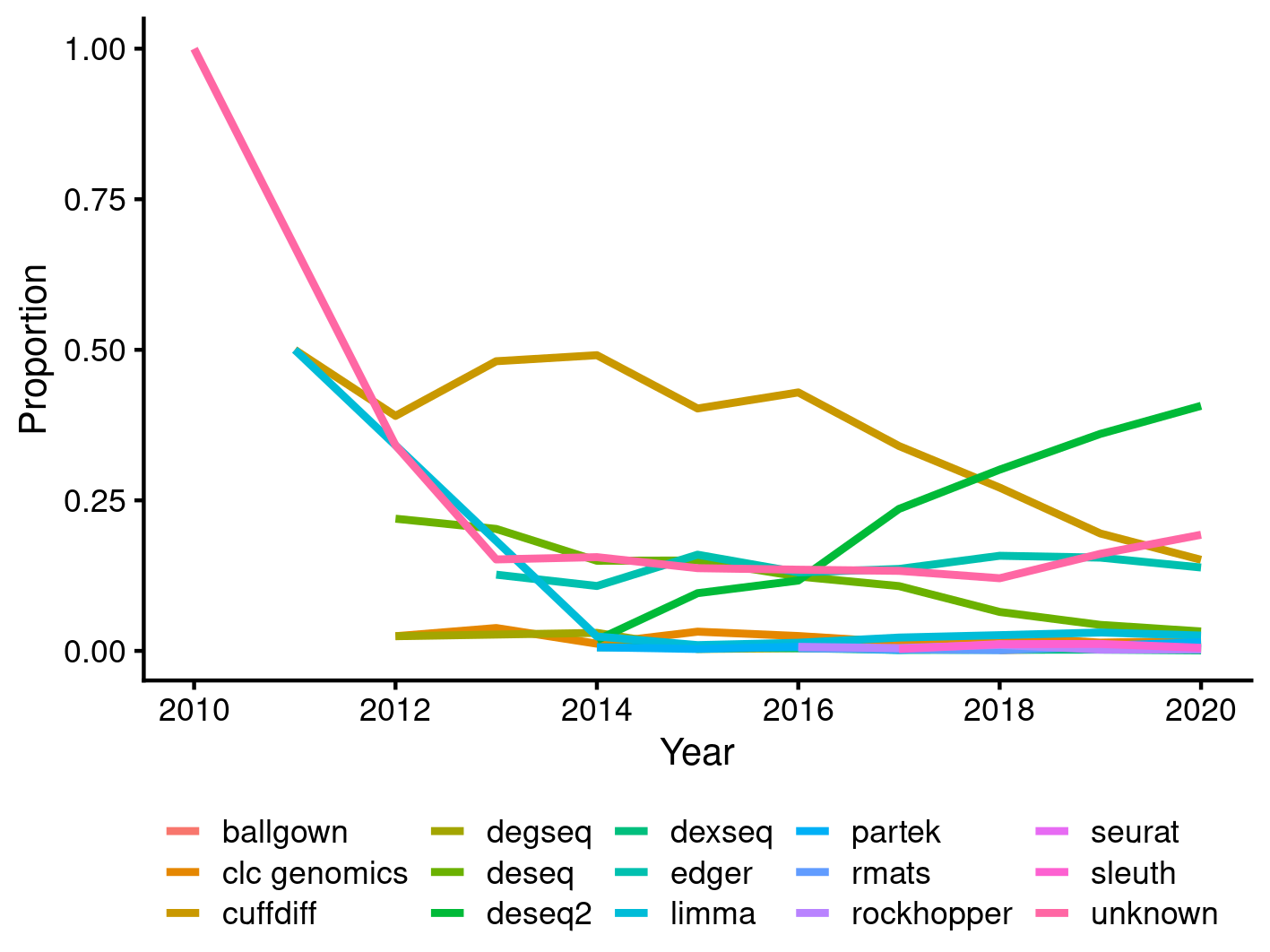

Supplement: S4 Fig — Y-axis shows the proportion of analysis platforms, x-axis shows publication year of GEO submission, N = 4,616. The data file is in S22 Data. (TIFF) [file pbio.3002007.s004.tiff]

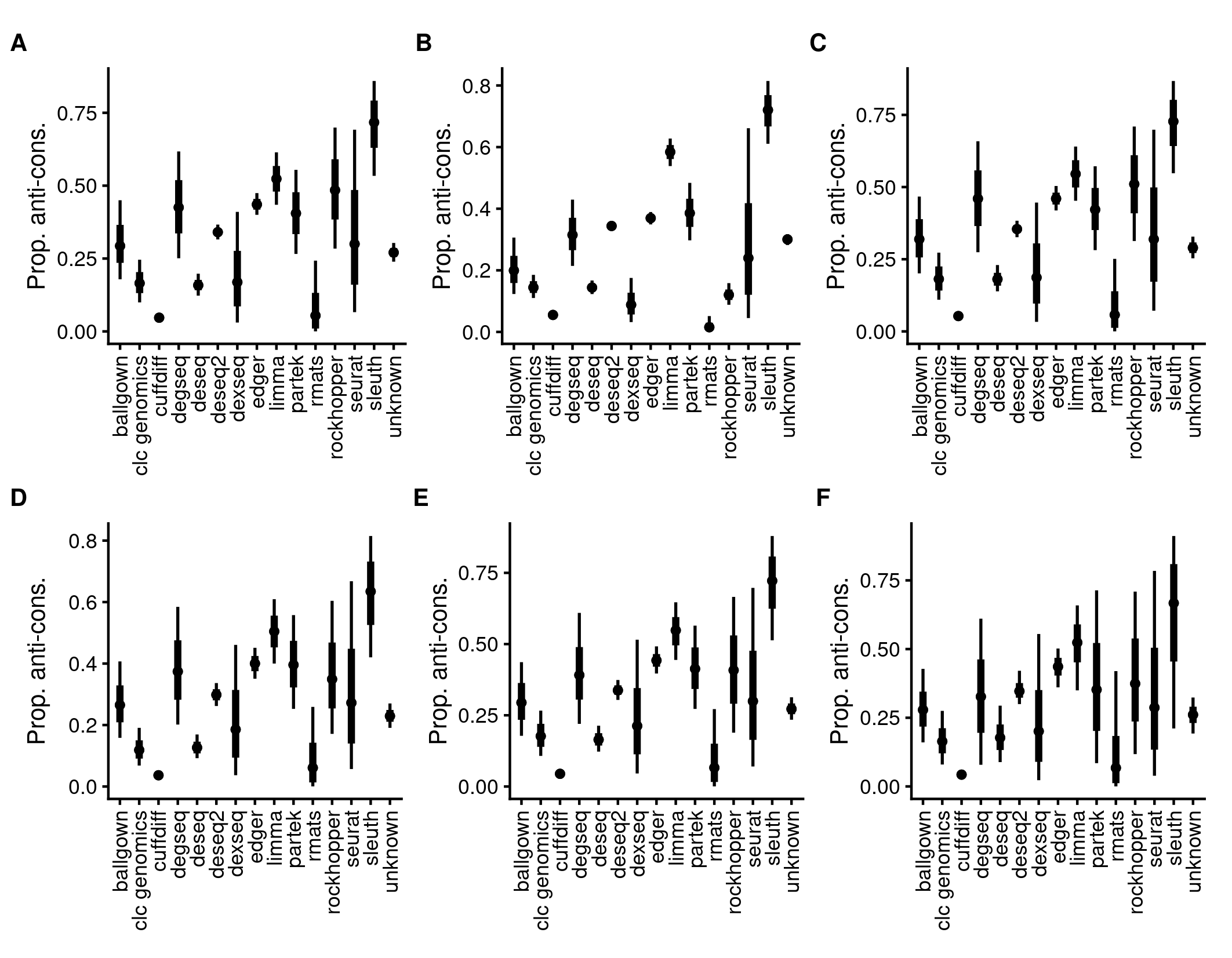

Supplement: S5 Fig — (A) Simple model [anticons ~ de_tool], N = 4,616. The data file is in S23 Data. (B) Simple model [anticons ~ de_tool] fitted on complete data, N = 14,813. The data file is in S24 Data. (C) Model conditioned on year of GEO submission [anticons ~ year + de_tool], N = 4,616. The data file is in S25 Data. (D) Model conditioned on studied organism (human/mouse/other) [anticons ~ organism + de_tool], N = 3,886. The data file is in S26 Data. (E) Varying intercept model [anticons ~ de_tool + (1 | model)] where “model” stands for sequencing instrument model, N = 3,778. The data file is in S27 Data. (F) Varying intercept and slope model [anticons ~ de_tool + (de_tool | model)], N = 3,778. The data file is in S27 Data. Points denote best fit of linear model. Thick and thin lines denote 66% and 95% credible interval, respectively. The model object related to panel A can be downloaded from https://gin.g-node.org/tpall/geo-htseq-paper/src/v0.1/models/anticons_detool.rds. The model object related to panel B can be downloaded from https://gin.g-node.org/tpall/geo-htseq-paper/src/v0.1/models/anticons_detool_all.rds. The model object related to panel C can be downloaded from https://gin.g-node.org/tpall/geo-htseq-paper/src/v0.2/models/anticons_year_detool.rds. The model object related to panel D can be downloaded from https://gin.g-node.org/tpall/geo-htseq-paper/src/v0.2/models/anticons_organism_detool.rds. The model object related to panel E can be downloaded from https://gin.g-node.org/tpall/geo-htseq-paper/src/v0.1/models/anticons_detool__1_model.rds. The model object related to panel F can be downloaded from https://gin.g-node.org/tpall/geo-htseq-paper/src/v0.1/models/anticons_detool__detool_model.rds. (TIFF) [file pbio.3002007.s005.tiff]

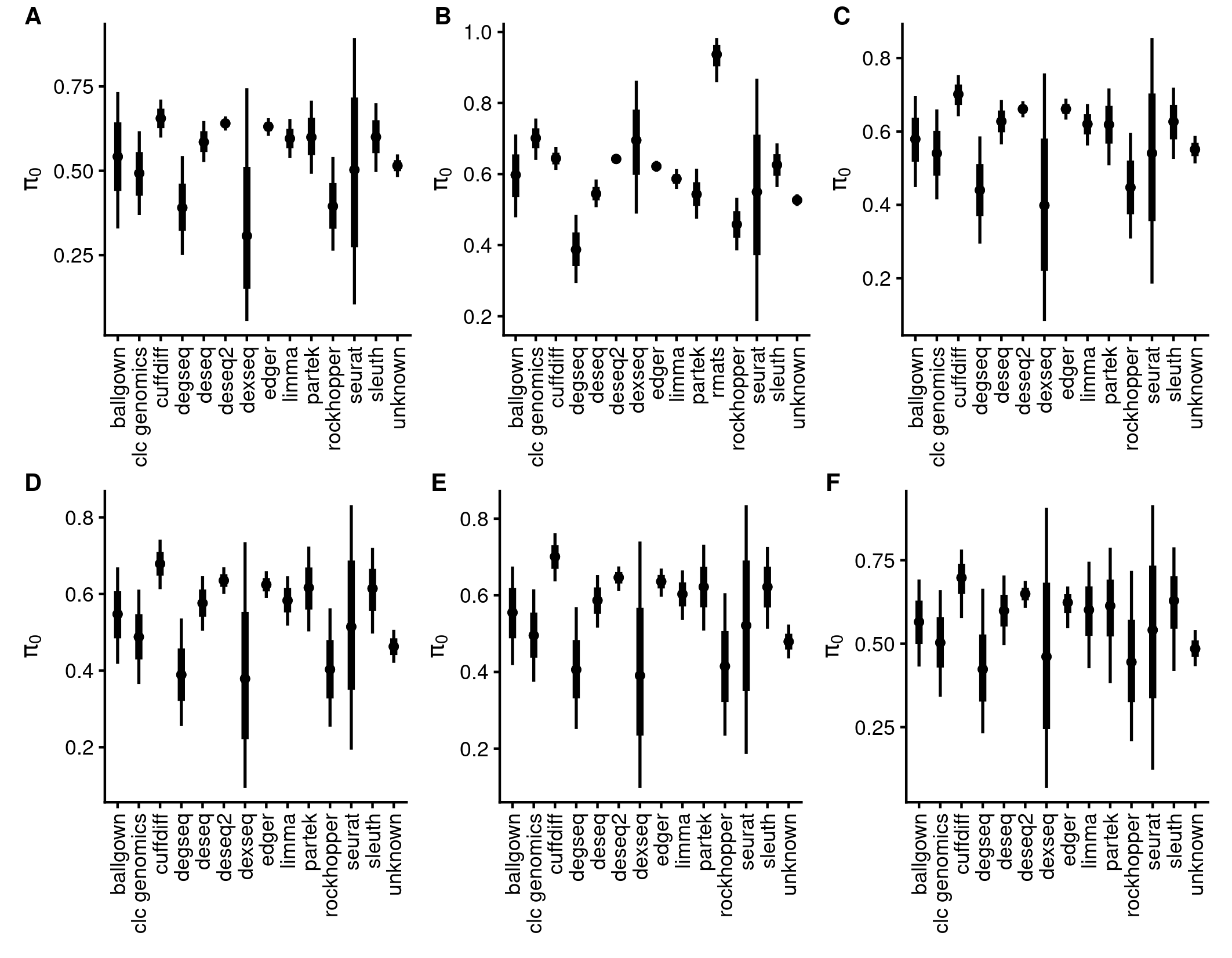

Supplement: S6 Fig — (A) Simple model [pi0 ~ de_tool] fitted on sample, N = 1,188. The data file is in S4 Data. (B) Simple model [pi0 ~ de_tool] fitted on complete data, N = 3,898. The data file is in S28 Data. (C) Model conditioned on year of GEO submission [pi0 ~ year + de_tool], N = 1,188. The data file is in S29 Data. (D) Model conditioned on studied organism (human/mouse/other) [pi0 ~ organism + de_tool], N = 993. The data file is in S30 Data. (E) Varying intercept model [pi0 ~ de_tool + (1 | model)] where “model” stands for sequencing instrument model, N = 959. The data file is in S31 Data. (F) Varying intercept/slope model [pi0 ~ de_tool + (de_tool | model)], N = 959. The data file is in S31 Data. Points denote best fit of linear model. Thick and thin lines denote 66% and 95% credible interval, respectively. The model object related to panel A can be downloaded from https://gin.g-node.org/tpall/geo-htseq-paper/src/v0.1/models/pi0_detool_sample.rds. The model object related to panel B can be downloaded from https://gin.g-node.org/tpall/geo-htseq-paper/src/v0.1/models/pi0_detool_full_data.rds. The model object related to panel C can be downloaded from https://gin.g-node.org/tpall/geo-htseq-paper/src/v0.2/models/pi0_year_detool.rds. The model object related to panel D can be downloaded from https://gin.g-node.org/tpall/geo-htseq-paper/src/v0.2/models/pi0_organism_detool.rds. The model object related to panel E can be downloaded from https://gin.g-node.org/tpall/geo-htseq-paper/src/v0.1/models/pi0_detool__1_model.rds. The model object related to panel F can be downloaded from https://gin.g-node.org/tpall/geo-htseq-paper/src/v0.1/models/pi0_detool__detool_model.rds. (TIFF) [file pbio.3002007.s006.tiff]

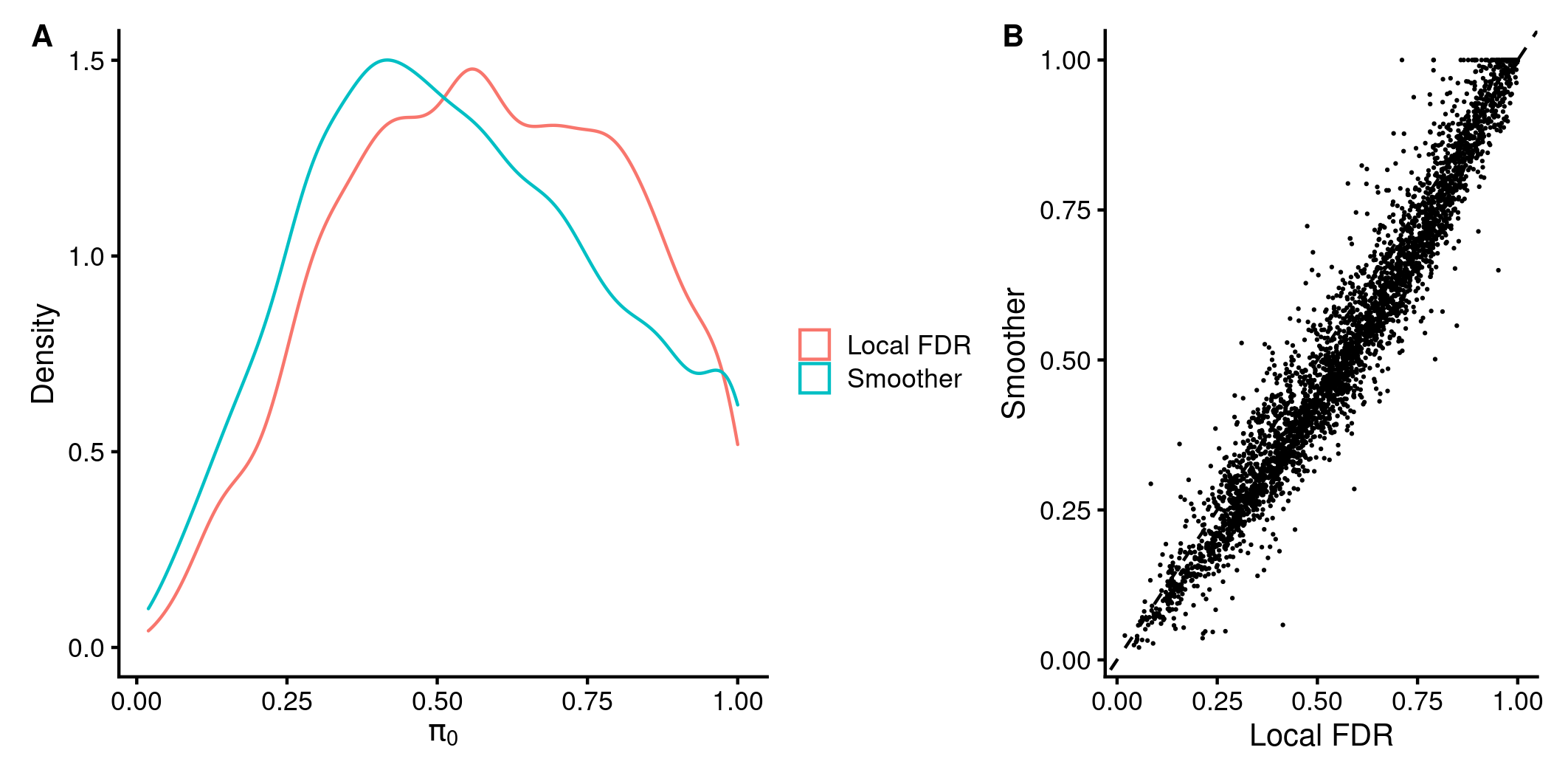

Supplement: S7 Fig — Local FDR method is from limma R package function propTrueNull. Smoother method is from q value R package. A, density histogram. B, scatter plot. Dashed line has intercept = 0 and slope = 1. The data file is in S32 Data. (TIFF) [file pbio.3002007.s007.tiff]

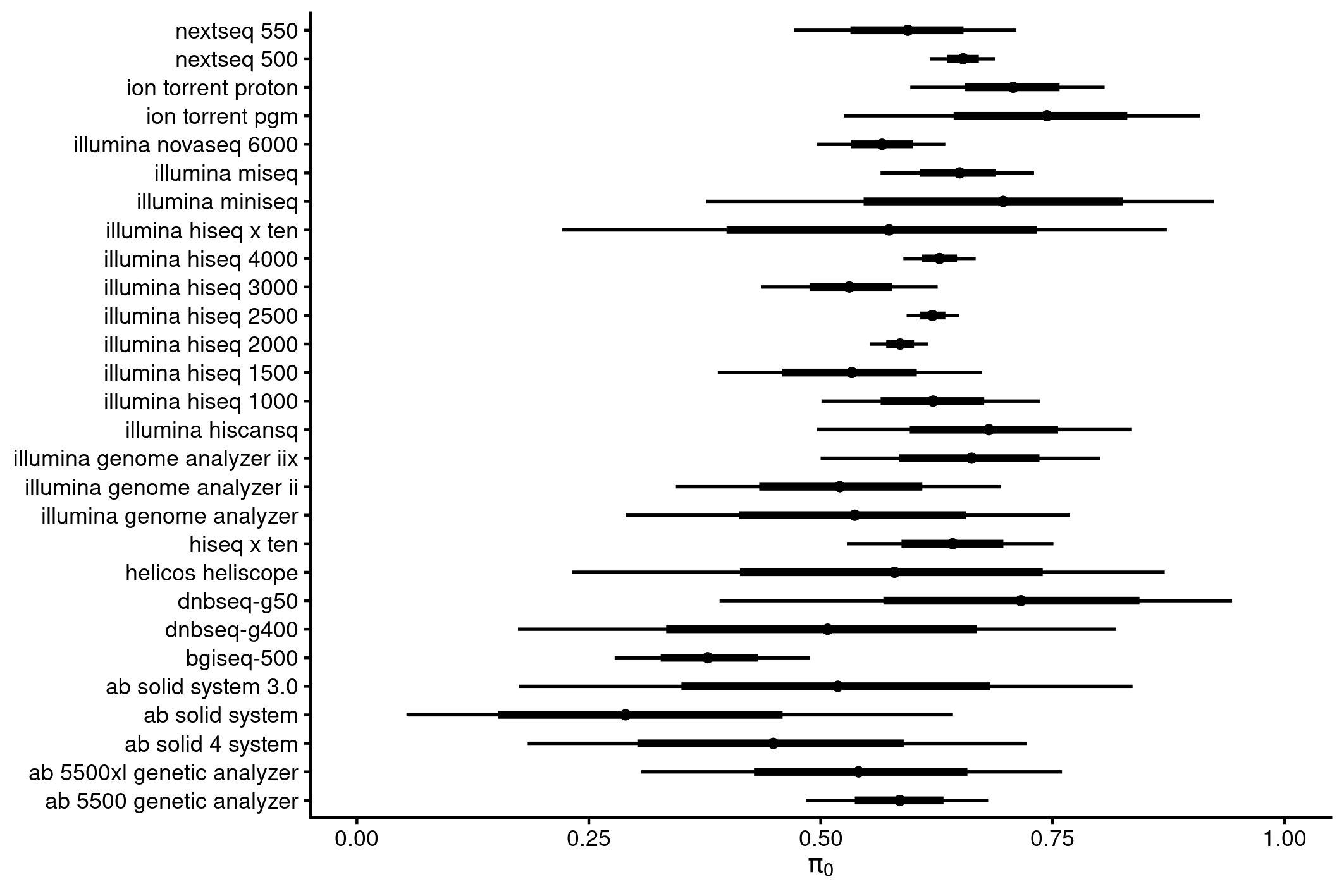

Supplement: S8 Fig — Points denote best fit of linear model ([pi0 ~ model], beta distribution, N = 959). Thick and thin lines denote 66% and 95% credible interval, respectively. The data file is in S33 Data. The model object related to figure can be downloaded from https://gin.g-node.org/tpall/geo-htseq-paper/src/v0.1/models/pi0__model.rds. (TIFF) [file pbio.3002007.s008.tiff]

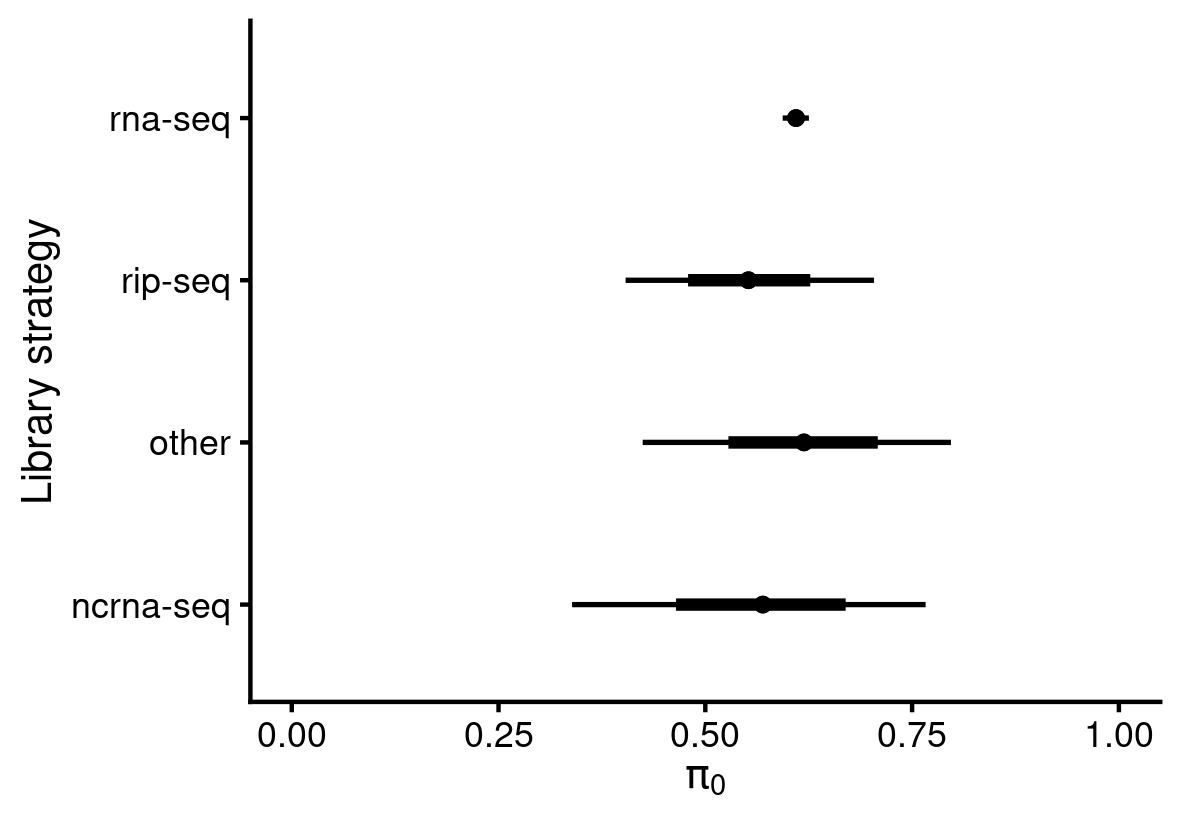

Supplement: S9 Fig — Points denote best fit of linear model ([pi0 ~ library_strategy], beta distribution, N = 959). Thick and thin lines denote 66% and 95% credible interval, respectively. The data file is in S34 Data. The model object related to figure can be downloaded from https://gin.g-node.org/tpall/geo-htseq-paper/src/v0.1/models/pi0__librarystrategy.rds. (TIFF) [file pbio.3002007.s009.tiff]

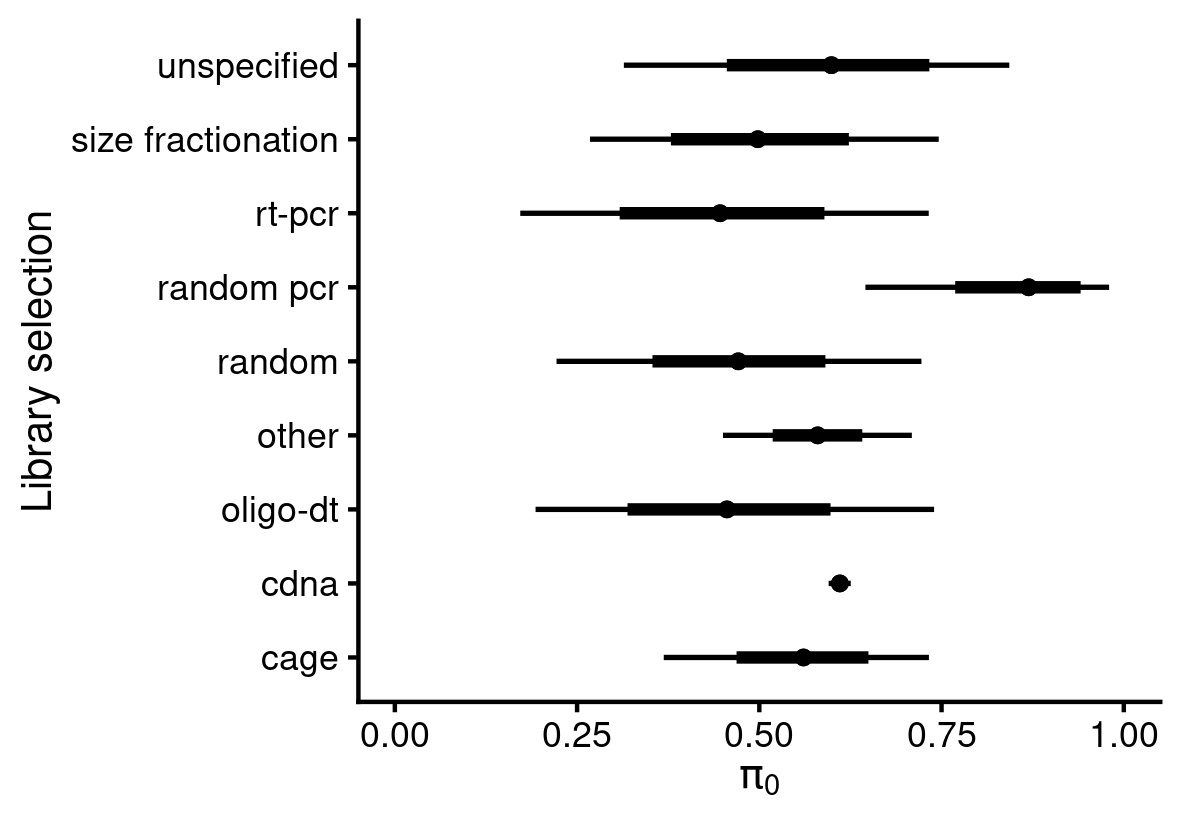

Supplement: S10 Fig — Points denote best fit of linear model ([pi0 ~ library_selection, beta likelihood], N = 959). Thick and thin lines denote 66% and 95% credible interval, respectively. The data file is in S35 Data. The model object related to figure can be downloaded from https://gin.g-node.org/tpall/geo-htseq-paper/src/v0.1/models/pi0__libraryselection.rds. (TIFF) [file pbio.3002007.s010.tiff]

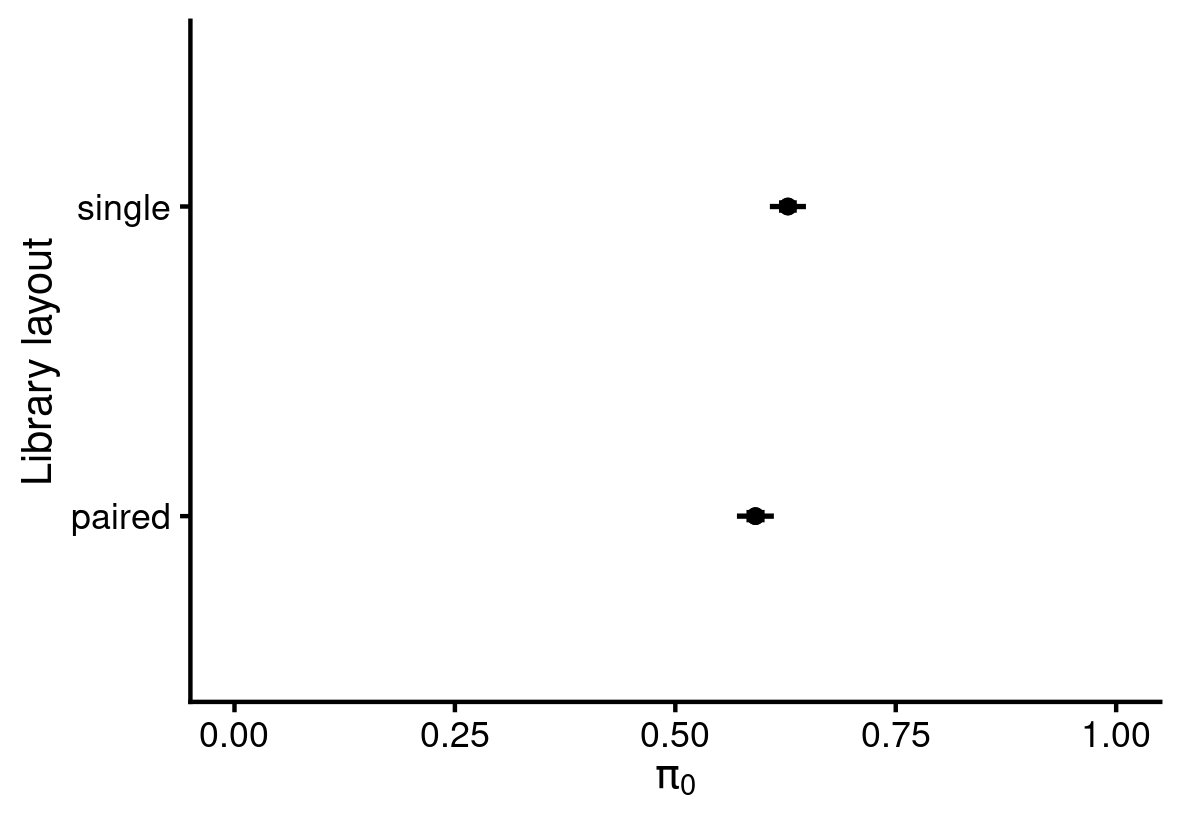

Supplement: S11 Fig — Points denote best fit of linear model ([pi0 ~ library_layout, beta likelihood], N = 959.). Thick and thin lines denote 66% and 95% credible interval, respectively. The data file is in S36 Data. The model object related to figure can be downloaded from https://gin.g-node.org/tpall/geo-htseq-paper/src/v0.2/models/pi0__1_librarylayout.rds. (TIFF) [file pbio.3002007.s011.tiff]

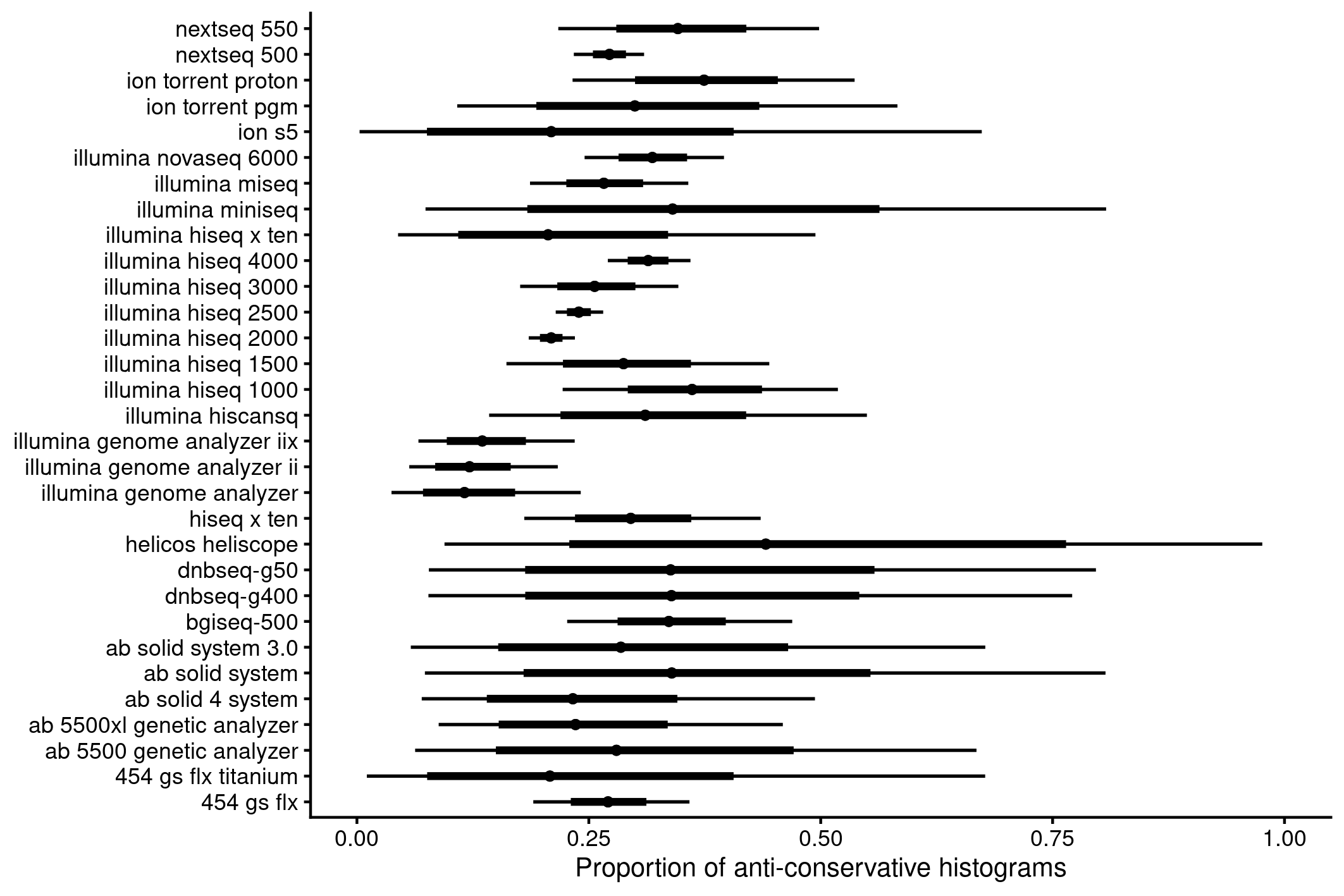

Supplement: S12 Fig — Points denote best fit of linear model ([anticons ~ model, bernoulli likelihood], N = 3,778). Thick and thin lines denote 66% and 95% credible interval, respectively. The data file is in S37 Data. The model object related to figure can be downloaded from https://gin.g-node.org/tpall/geo-htseq-paper/src/v0.2/models/anticons__1_model.rds. (TIFF) [file pbio.3002007.s012.tiff]

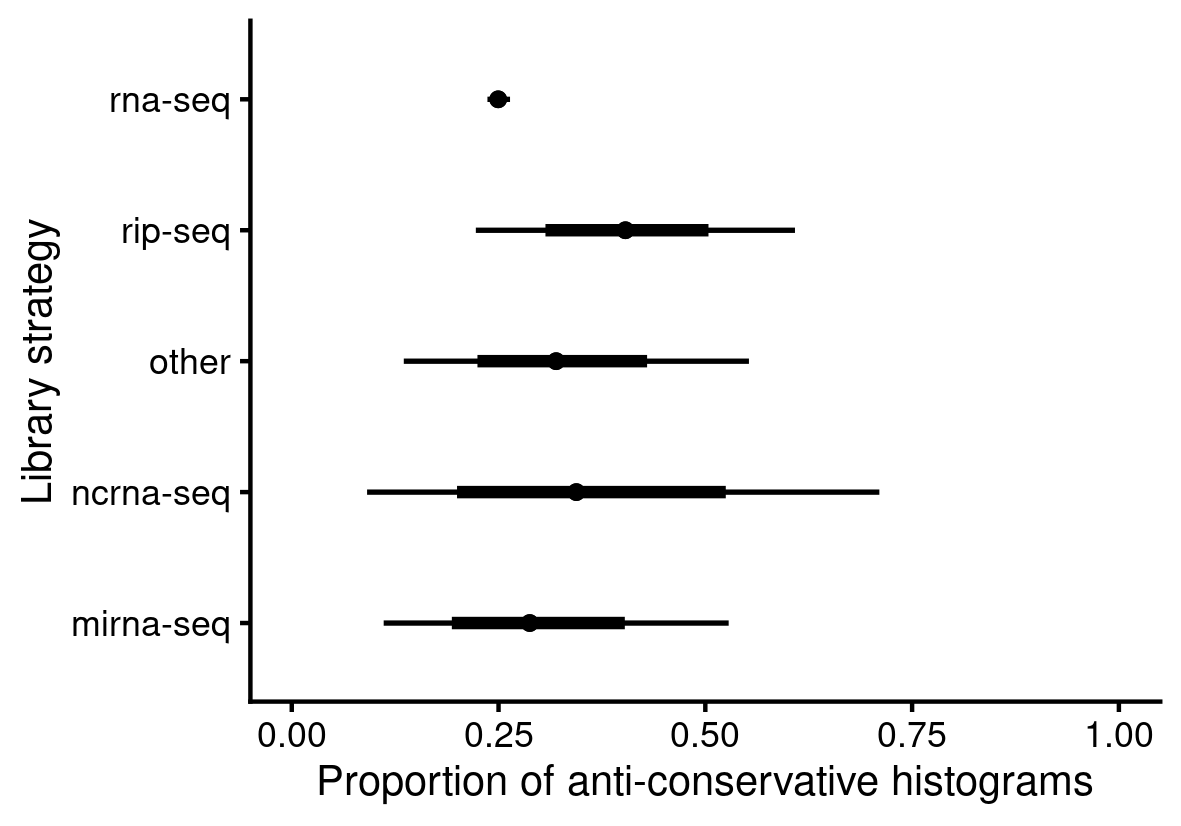

Supplement: S13 Fig — Points denote best fit of linear model ([anticons ~ library_strategy, bernoulli likelihood], N = 3,778). Thick and thin lines denote 66% and 95% credible interval, respectively. The data file is in S38 Data. The model object related to figure can be downloaded from https://gin.g-node.org/tpall/geo-htseq-paper/src/v0.2/models/anticons__librarystrategy.rds. (TIFF) [file pbio.3002007.s013.tiff]

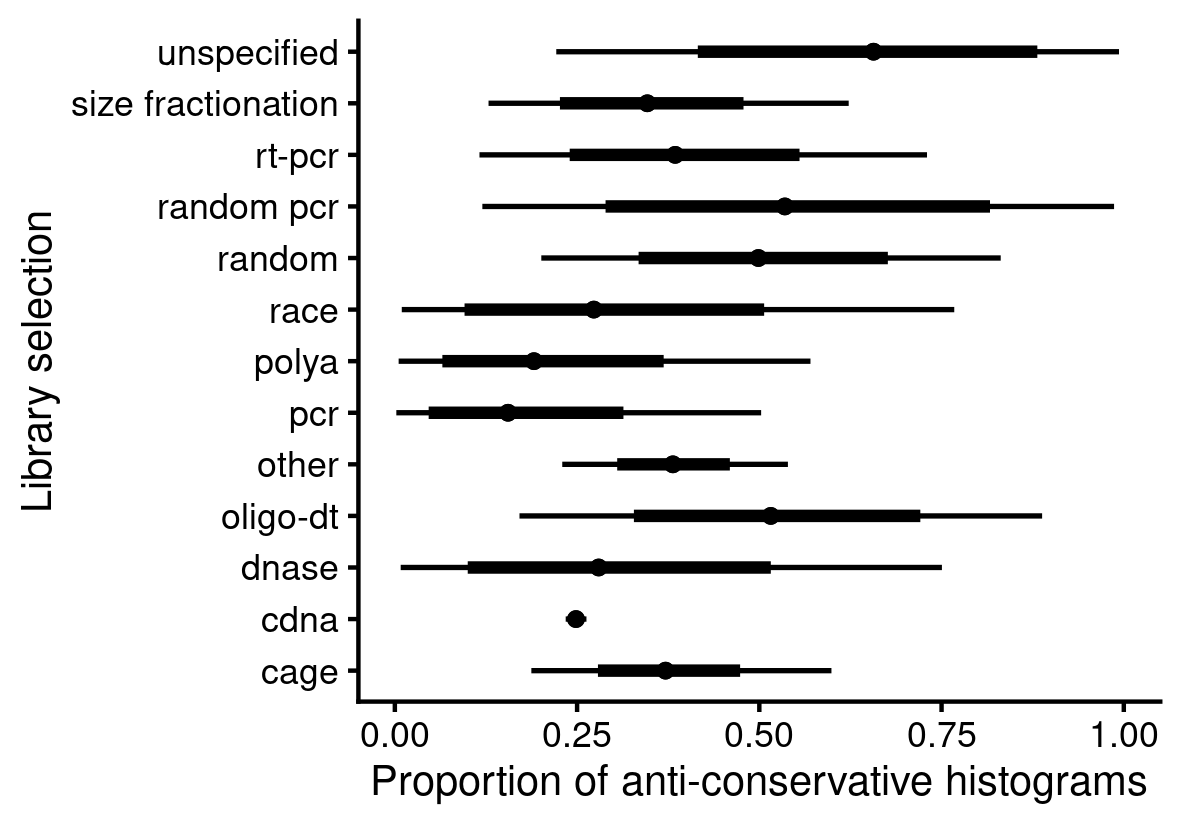

Supplement: S14 Fig — Points denote best fit of linear model ([anticons ~ library_selection, bernoulli likelihood], N = 3,778). Thick and thin lines denote 66% and 95% credible interval, respectively. The data file is in S39 Data. The model object related to figure can be downloaded from https://gin.g-node.org/tpall/geo-htseq-paper/src/v0.1/models/anticons__libraryselection.rds. (TIFF) [file pbio.3002007.s014.tiff]

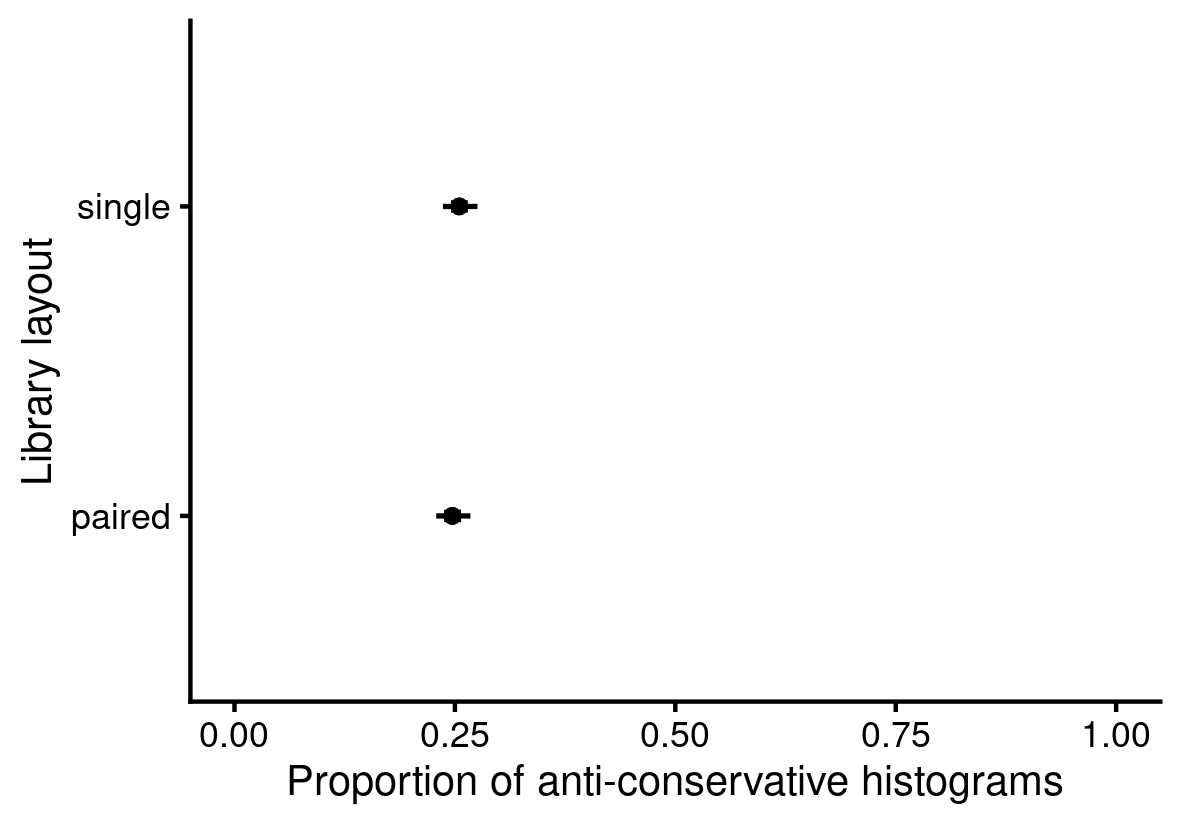

Supplement: S15 Fig — Points denote best fit of linear model ([anticons ~ library_layout, bernoulli likelihood], N = 3,778). Thick and thin lines denote 66% and 95% credible interval, respectively. The data file is in S40 Data. The model object related to figure can be downloaded from https://gin.g-node.org/tpall/geo-htseq-paper/src/v0.1/models/anticons__librarylayout.rds. (TIFF) [file pbio.3002007.s015.tiff]

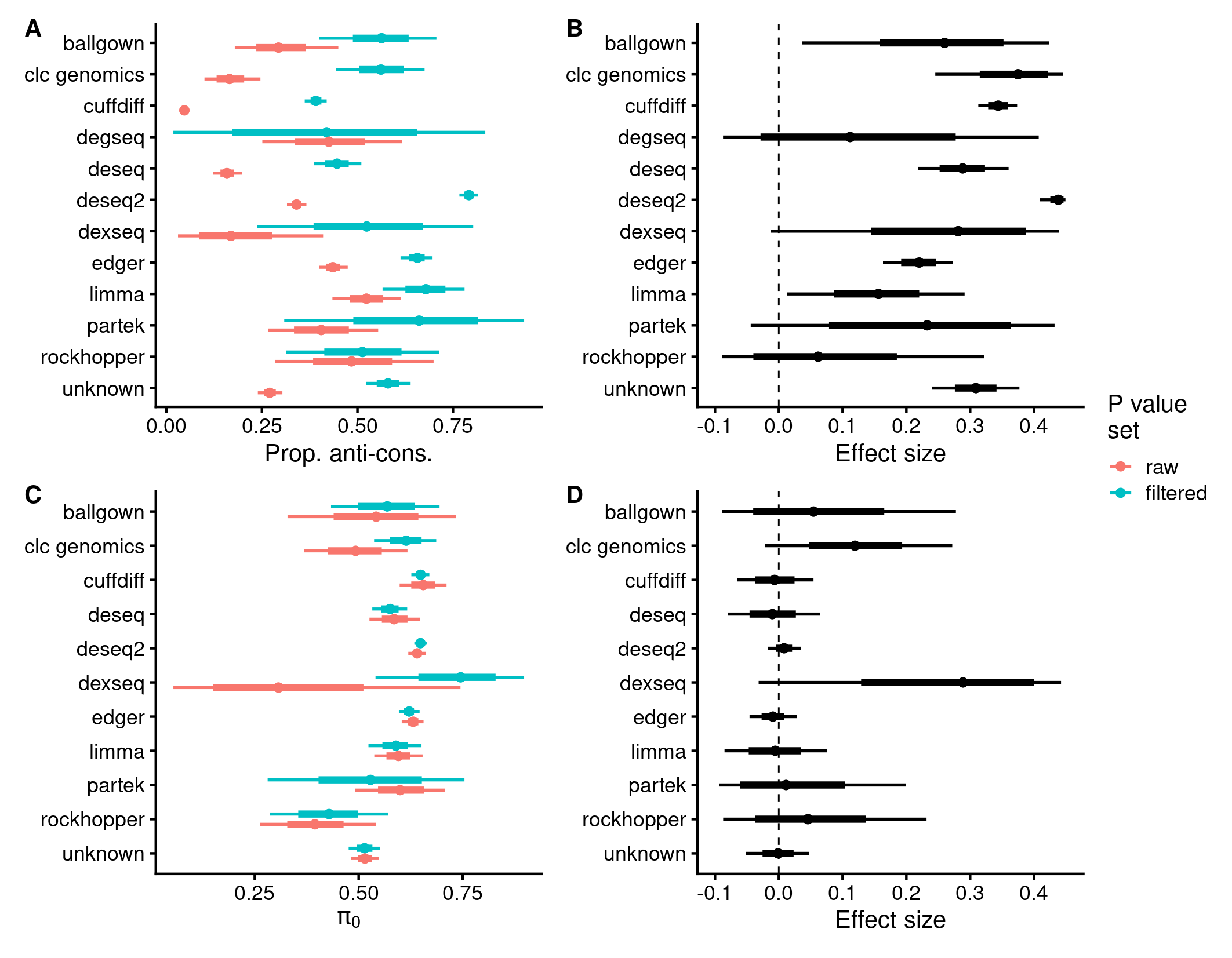

Supplement: S16 Fig — (A) Anti-conservative p-value histogram proportions in raw and filtered p-value sets for DE analysis programs. Raw p-value data is the same as in S5A Fig. Filtered p-value data is from a simple Bernoulli model [anticons ~ de_tool], N = 3,426. The data files are in S13 Data and in S14 Data (for raw data). (B) Effect size of low-count feature filtering to proportion of anti-conservative p-values. The data files are in S13 Data and in S14 Data (for raw data). (C) π0 estimates for raw and filtered p-value sets. Raw p-value data is the same as in S6A Fig and filtered p-value data is from the beta model [pi0 ~ de_tool], N = 2,042. The data files are in S15 Data and in S16 Data (for raw data). (D) Effect size of low-count feature filtering to π0. The data files are in S15 Data and in S16 Data (for raw data). Points denote model best fit. Thick and thin lines denote 66% and 95% CIs, respectively. (TIFF) [file pbio.3002007.s016.tiff]

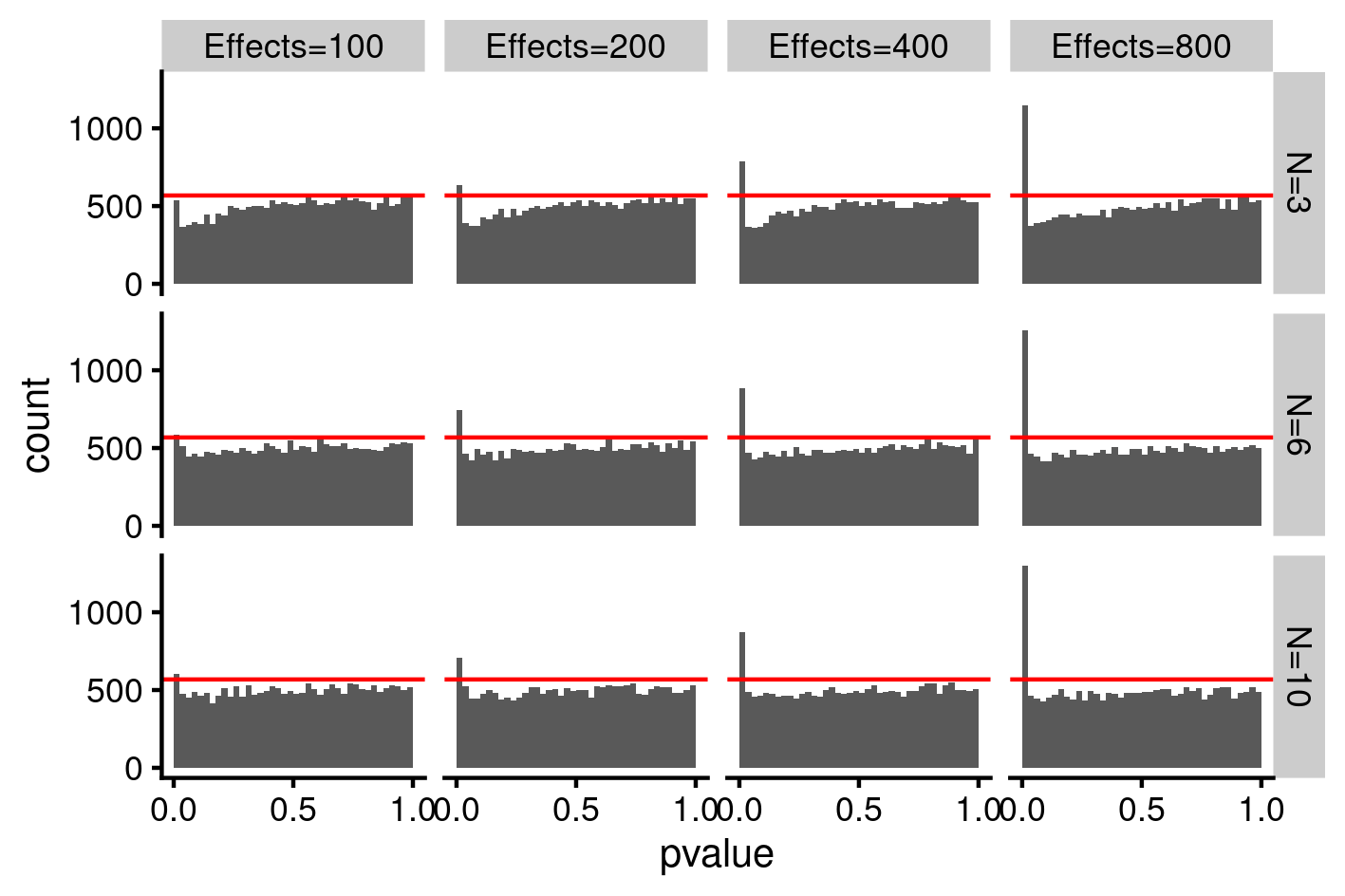

Supplement: S17 Fig — RNA-seq data was simulated with polyester R package on 20,000 transcripts from human transcriptome using grid of 3, 6, and 10 replicates and 100, 200, 400, and 800 effects for 2 groups. Fold changes were set to 0.5 and 2. Differential expression was assessed using DESeq2 R package using default settings and group 1 versus group 2 contrast. Effects denotes in facet labels the number of true effects and N denotes number of replicates. Red line denotes QC threshold used for dividing p histograms into discrete classes. Code and workflow used to run these simulations is available on Github: https://github.com/rstats-tartu/simulate-rnaseq. Raw data of the figure is available on Zenodo https://zenodo.org with doi: 10.5281/zenodo.4463803. (TIFF) [file pbio.3002007.s017.tiff]
