# Supplementary material for: A field-wide assessment of differential expression profiling by high-throughput sequencing reveals widespread bias
Source: PLoS Biol. 2023 Mar 2;21(3):e3002007. doi: 10.1371/journal.pbio.3002007 (PMC10013925; doi:10.1371/journal.pbio.3002007)
Supplement: Attachment — Submitted filename: response to reviewers.docx [file pbio.3002007.s058.docx]

Here we answer the referees' questions one by one.

Reviewer #1:
The importance of studies that examine the reliability of scientific findings cannot be overstressed. However, while notwithstanding the effort and scale of the analysis presented in this study, I find that, in its current form, its impact and significance are limited. The study points to a widespread issue in the field of DE analysis. This is no doubt an interesting observation. However, to my view, this observation on its own is not sufficient to make it important/significant/useful.

Let's put it this way. Suppose I do an experiment, and somebody rightly points out that the experiment cannot be analyzed or interpreted properly. Clearly, I cannot ignore this, even if I have no immediate idea how to fix it. It is up to me to find a solution, and there is no way I will go on as if nothing happened. So, how can I not find this important/significant/useful?

I would regard this observation as a first step of detecting a (potentially) important problem in the field.

We have now taken a step further in pinpointing our main results to the failure to control FDR. From our resubmission, there should be little doubt that the problems we point out are real (not just statistical curiosities) and pervasive. They are also seen in recent methodological studies as a widespread failure of existing DE analysis platforms to control FDR. So it is not just us anymore; the evidence is coming in from different directions.

But there are three critical subsequent steps that are completely missing in the current study: (1) What are the consequences of the problem? Does it lead to serious biological misinterpretations of the data? Previous technical studies in this field did show how biases lead to specific functional misinterpretations.

These consequences we now put as follows (in the Discussion):

*Collectively, our real-world study and the aforementioned simulation studies indicate that the field is largely built on analyzing low-power experiments, which are unlikely to identify true effects, but still tend to over-estimate the effect sizes (of both true and false DE-s) of any statistically significant results, while presenting an unknowable number of non-existing effects as statistically significant. It is hard to imagine a worse state of affairs or that this would not have a substantially detrimental impact on the quality of relevant scientific conclusions.*

To further illustrate the consequences of bad p-values, we can look at a rare published study where two different DE tests were used on the same data. Hirota et al. Dev Cell 2018 47:645-659.e6. PMID: 30393076 (GEO Series GSE107671) analyzed the same DE RNA-seq data in parallel with two quite similar tests, the likelihood ratio test (lrt) and the Wald test. The p-value histograms and scatter plot of the p values look like this:

They calculated the q values from both (and then used only the Wald test results without comment). The LRT test resulted in 0 genes q < 0.1 (lowest q = 0.107), while the Wald test resulted in 1411 genes q < 0.1 (lowest q = 10^-35^), a.k.a. 6% of the genes. Thus, at least in this study, the consequences of a misshapen p-value distribution are very substantial indeed.

(2) What causes this bias/issue?

There is no simple way to address this question. Moreover, we see no reason to suppose a single cause exists. Instead, our partial rescue of p-value histograms due to additional filtering suggests multiple causes. We think each RNA-seq experiment likely has its own mix of biases emanating from the interaction of a particular experimental design with a specific choice of analytic workflow. This question is unsuitable for a meta-science approach but needs individual treatment in the context of a specific experiment.

(3) How to correct/alleviate this issue? 

The last chapter of the Discussion, subtitled "Considerations for improvement of best practices", tries to answer this. Briefly, we see each RNA-seq experiment as needing individual treatment. Therefore, instead of offering a single winning workflow, the user should be given a toolbox from which she can combine a good workflow suitable for her experiment. Of course, as long as the power of experiments is not improved, it is unlikely that any method of analysis really works (as there has to be enough information present to analyze). Also, very recent p-value-free methods could be useful as they do not depend on parametric assumptions and yet have reasonable power to detect effects – but only time can tell.

These three subsequent steps are discussed in the Discussion of this study. As such the study indeed gives, as the authors put it, "a birds-eye view" on the DE field. The impact and utility of this are, to my view, limited.   To make it a high-impact study speculative discussions should be replaced by solid analyses.

We believe that the referee has it backwards. To make the field of RNA-seq anything but a collection of speculative analyses, much work should be done to develop better investigative techniques. This is the responsibility of the whole field, not our meta-science study. Our work shows the need for such a considerable effort and perhaps offers a few hints on how to start. It is not our job to cure the patient, just to diagnose her.

Few examples from the Discussion:
*	Line 276: "Interestingly, limma and edgeR use very different distributional models for DE testing, supporting the notion that it might be data preprocessing, rather than the statistical test, that has the most impact on the success of DE analysis". 

-	Please demonstrate this. Show how changing preprocessing (beyond filtering low expression) affect the issue.

As per above (and our Discussion), many have tried to do a comparative analysis of different workflows, but to little effect in understanding what works and what does not. Again, the reason might be that different experiments could require different analyses. To repeat these studies on a case-by-case basis would require the implementation (and description in the paper) of a completely different methodology. We feel that to publish all this in a single paper would be unkind towards the reader and would dilute the impact of our large-scale approach rather than increase it.

*	 Line 403: "This raises the possibility that nearly all experiments, which should have resulted in uniform distributions, were somehow shifted into other distributional classes".

-	The authors can thoroughly check this by running different workflows to compare replicate samples in many datasets. Do they observe the same problem and same association with DE tool / processing pipeline?

To manually run hundreds of workflows to extract possible uniform p-value distributions from the noise does not sound like a good investment in terms of time or resources. 

*	Line 410. "In principle, a low PI0 could reflect true differential expression as a causal response to experimental treatment, or it could be an artefact of suboptimal data analysis."

-	Again, what pi0 values do you get if you systematically apply DE analysis on replicate samples using the different tools and preprocessing pipelines?

That is a good question, but for the people doing individual RNA-seq experiments. This would constitute a reasonable robustness analysis, which we heartily recommend. 

*	Line 417. "which suggests that the low pi0-s in our dataset are indicative of problematic analytic workflows." 
-	What step in the pipeline? How to alleviate?

We have no idea. Again, it is not for us to cure the patient.

*	Line 430. "it seems likely that many or most of the low pi0 experiments represent technical failures, most likely during data normalization." 

-	This point should be strongly demonstrated rather than speculated on.

This demonstration would require a very work-intensive case-by-case study, which is better left to methodologists.

*	Line 436. "How much this level of technical malpractice influences the quality of the actual scientific conclusions - which is what really matters in the end - is an open question"

-	Indeed. Without some exploration of this question, the impact of the study is of limited importance.

We have now removed the whole paragraph containing the sentence to tighten our Discussion. The importance is now addressed in the first part of Discussion, and as mentioned before, we have a much stronger argument to make in this regard.

*	Line 438. "but one cannot but assume that this influence is substantial, and in need of corrective action."

-	Agree. But a much deeper analysis should be done about the corrective actions.

See our answer to the previous comment.

*	I spotted one tangible recommendation - filtering out lowly expressed genes. This is not a novel recommendation, and it is made by most of the popular DE tools in the field.

This is an important point. Although most DE analysis tools remove low counts more or less automatically, our work clearly shows that this is not enough and that the scientist should take more control over the pre-filtering. Let it be noted that the level of pre-filtering will also depend on the expression levels of proteins of scientific interest to the researcher (as will the sample size and power). Also, in the revised manuscript, we show that the fraction of anti-conservative p-value histograms is strongly associated with sample sizes in the range of N=2 to N=5 (and the vast majority of empirical N-s are in that range, being mostly 2 and 3). Thus, increasing the N would be another obvious recommendation, as well-powered experiments are (maybe) more amenable to analysis with existing methods. We apologize if the referee has heard that one before, but something should be done about it at some point. 

A typo: Line 391: refers to Fig S18. 
We corrected this.

Reviewer 2

Major points:
1. The DE analysis tools used in different studies/datasets were not well clarified. In Figure 2, there are only five tools: limma, cuffdiff, deseq, edger, and unknown. I do not think only 4 methods (except "unknown") were used in all those studies.

We did a thorough reanalysis and now have identified 5 major platforms (DESeq2, Cuffdiff, DESeq, EdgeR, and limma) and 9 minor ones (ballgown, clc genomics, degseq, dexseq, partek, rmats, rockhopper, seurat, and sleuth). This turned out to be useful, as we can now more clearly see that more recent platforms work better than the older ones (regardless of program updates). Otherwise, the higher granularity of analysis did not change our conclusions.

If "unknown" means all other methods, it should be named as "others" instead of "unknown".

“Unknown” means that we were unable to determine the DE analysis platform that was used to produce these p-values. We tried from full text articles, GEO summaries, GEO supplementary file names, p-value set names (people manually edit DE analysis results/top table variable names by appending contrast info or DE analysis tool info), and from variable name pattern in DE analysis results tables, which are usually tool-specific.

Besides, DESeq and DESeq2 use different models and should be considered two tools instead of one. The authors should replace "deseq" with a more specific names ("DESeq", "DESeq2" or "DESeq/DESeq2").

We did this.

2. As the authors discussed, the non-parametric methods may be a better choice in large-sample-size differential analysis. It would be great if the authors can look into some studies/datasets using non-parametric DE analysis methods. Although text mining may be difficult, the authors can search for the keywords "Wilcoxon" and "rank-sum test" in the published papers.

We tried and failed, using text mining to find a reasonable quantity of such studies. The non-parametric methods require relatively large sample sizes (N-s of at least around 10) for appreciable power (Ge, et al. Genome Biol 22, 288 (2021). Our analysis of sample sizes found very few suitable candedates for such tests.

3. The sample sizes of the datasets can also affect the performance of different DE tools. The sample-size effect on p-value distributions should also be evaluated.

This is an excellent point. We did that and found (1) that the studies are generally underpowered (by combining empirical sample sizes with power simulations with realistic levels of variation and effect sizes) - Fig. 4; (2) that there is a strong effect of N on the proportion of anti-conservative p-value histograms, but only between sample sizes of one and five - Fig. 5; (3) that there is no correlation of N with pi0-s, although by statistical theory and by simulation such a correlation should exist - Fig. 6. This adds further weight to our conclusion that the observed pi0 distribution is indicative of serious analytical problems in the field, even when the p-value distributions are good.

4. The filtering step was not specified. Although we do not need to find an optimal filtering procedure in this manuscript, the authors should at least show what the threshold is, and how the threshold is decided. If multiple thresholds were tried, the results should be compared. If different thresholds were used for different species, which should be the case because different species have different numbers of genes, the different thresholds should also be listed.

Filtering thresholds are described in Methods subsection “NCBI supplementary file processing”. Specifically, we used following thresholds: basemean=10, logcpm=1, rpkm=1, fpkm=1, aveexpr=3.32 to obtain filtered p-value sets during supplementary file processing. As different tools use different transformations of read counts, we tried to choose thresholds that end up in the same ballpark. However, there is no single best threshold, and as any modeling exercise, DE analysis is iterative and should include visual inspection of the results to identify best threshold for particular analysis. Indeed, a recent paper shows that there is no single optimal procedure and proposes an adaptive method for filtering (Zehetmayer, S., et al. Bmc Bioinformatics 23, 388 (2022).) We included this in the Discussion.

5. The analysis of pi_0 is not informative or convincing. The estimated pi_0's are not the true proportions of nulls but just intermediate quantities, which are used to increase the power of identifying DE genes.

Questioning the ontological status of pi0 is a good point, on which we weren't clear enough in the MS. Our goal is not to estimate the true proportion of zero magnitude effects (as if there even was such a thing, except zero). Instead, we show that pi0s, as estimated from the actual p-value distributions, are not indicative of good experimental practices – if most experiments would have really resulted in almost half of the features being DE in some biologically meaningful fashion, then we know that most of these experiments were designed (and analyzed) improperly. Also, we now show that the expected dependence of estimated pi0 on the sample size is simply not there. Thus our improved analysis of pi0-s is informative in that it convincingly shows that even the anti-conservative p-value distributions are problematic for downstream analysis. So we really use the pi0 as a single-number summary of the p-value distribution. 

Why not focusing on the proportion of DE genes (e.g. genes with FDR<0.05), which is a more straightforward statistic.

We did this analysis, but do not include it here. In the absence of trust that p-value sets culled from GEO produce pi0-s, which reflect the actual proportion of H0-s, these results are hard to interpret. They do not change our conclusions but would needlessly complicate the already complicated paper.

5. In the results part, cuffdiff is different from other DE tools in almost all the analyses. The authors should provide some explanation for this phenomenon.

Cuffdiff seems to underperform in many (but not all) published workflow comparisons. In our data, it leads to very few anti-conservative p value sets, but those cuffdiff sets that are anti-conservative give, on average, the highest pi0-s. As our analysis sees the actual use of methods in the field, it reflects sociology as well as methodology. For this reason, we are loath to conclude from our results anything about the supposed inferiority of Cuffdiff. It may be that the very high popularity of Cuffdiff reflects its simplicity of use, which attracts less competent users who have already harmed their experiments in some way before reaching the analysis stage. From our data, we see that people who do N=1 and N=2 experiments flock to Cuffdiff (but then, thankfully, not every program makes it easy to analyze N=1 experiments). On the other hand, people who do N=5+ experiments slightly prefer limma but seem to avoid Cuffdiff (even in absolute numbers, Cuffdiff has only 22 N = 5+ experiments, while the 13.6 times less popular limma has 45). So, we can provide a description but probably should not offer an explanation here.

6. In Line 175, the authors wrote, "the increase in the fraction of anti-conservative histograms is accomplished by decreases mostly in the class of other". However, Figure 2A also showed apparent decreases in the class of bimodal and conservative. The authors should provide quantitative measurements to support their description or change it.

As we reanalyzed this more granually, we accordingly changed the text. The measurements are quantitative with 95% CI-s.

New text: *Multinomial hierarchical logistic regression indicates that most differential expression (DE) analysis tools exhibit temporal increases of anti-conservative p-value histograms, except for cuffdiff, which has the opposite trend, and glc genomics and deseq, where a clear direction could not be identified (Fig. 2A, S3 Fig.). The increase in the fraction of anti-conservative histograms is accomplished by decreases mainly in the class "other" and "bimodal", depending on the DE analysis tool.*

7. In Line 236, the results which support the conclusion that using another method does not change the results should be provided.

We did so and rewrote the text to describe the new supplementary figure:

*Recalculating the π_0-s with a global FDR algorithm [28] did not lead to a substantially different π_0 distribution. However, there appears a small directional shift of the distribution, whereby the mean π_0 is shifted from 0.58 in the local FDR method to 0.54 in the global FDR method (S7 Fig.).*

8. The authors concluded negative results from Line 237 to 241, but I do see associations with variables like library layout (Fig. S11). The statistical tests and corresponding p-values which support the conclusions should be included.

On this technical point, we respectfully disagree. Yes, there is an effect in S11 Fig., and yes, the p-value would be very small (as can be seen from the CI-s), but the interpretation of this p-value could be misleading. As the effect is small (only a couple of percentage points, where an important effect size would be maybe 10-20 percentage points) and the N is quite big, then a small p-value would mostly be a comment on the N rather than on the effect size.

We formally conclude in the Results: "As there is a strong association between both π0 (S7-10 Fig.) and proportion of anti-conservative p-value histograms with DE analysis tool …, we further checked for, and failed to see, similar associations …. These ~~negative~~ results support the conjecture of specificity of the associations with DE analysis tools.".

In a scientific sense, a very small effect is as good as no effect, and the fact that we have a small but statistically significant effect just tells us that although we have some bias in our analysis, it is small enough to be safely ignored.

Minor points:
1. This manuscript only looked at the p-value-based DE tools. However, some DE tools that do not rely on p-values have been proposed (e.g. <https://genomebiology.biomedcentral.com/articles/10.1186/s13059-021-02506-9>) These methods can get rid of the problems regarding problematic p-values and are worth Discussion.

We are eternally grateful for this suggestion, because reading this outstanding paper allowed us to rethink our conclusions and more strongly interpret our pi0 findings through the lack of FDR control. Otherwise, as suggested by the referee, we discussed the p-value-free methods in the Discussion.

2. The discussion section is very long. The authors may consider using subheadings.

We did so.

3. Almost all main figures have overlaps with the system-generated figure numbers, making the figures hard to read. The authors should increase the figure margins.

We corrected this.

4. Some sentences are too long to understand (e.g., the one that begins at Line 249). The authors should consider rewriting them.

Thank you for pointing this out, we did what we could.

__________________________________________________
